# Mechanisms of Xiaozheng decoction for anti-bladder cancer effects via affecting the GSK3β/β-catenin signaling pathways: a network pharmacology-directed experimental investigation

**DOI:** 10.1186/s13020-023-00818-5

**Published:** 2023-08-22

**Authors:** Jingming Zhuang, Jiahang Mo, Zhengnan Huang, Yilin Yan, Zeyi Wang, Xiangqian Cao, Chenkai Yang, Bing Shen, Fang Zhang

**Affiliations:** 1 Department of Urology, Shanghai General Hospital, Shanghai Jiao Tong University School of Medicine, Shanghai, China; 2 Department of Gynecology, Obstetrics and Gynecology Hospital, Fudan University, Shanghai, China; 3 Department of Urology, Tongji Hospital, School of Medicine, Tongji University, Shanghai, China; 4 Department of Urology, Shanghai General Hospital Affiliated to Nanjing Medical University, Shanghai, China

**Keywords:** Xiaozheng decoction, Bladder cancer, Network pharmacology, GSK3β/β-catenin, Apoptosis

## Abstract

**Purpose:**

The combination of Xiaozheng decoction with postoperative intravesical instillation has been shown to improve the prognosis of bladder cancer patients and prevent recurrence. However, the mechanisms underlying the efficacy of this herbal formula remain largely unclear. This research aims to identify the important components of Xiaozheng decoction and explore their anti-bladder cancer effect and mechanism using network pharmacology-based experiments.

**Methods:**

The chemical ingredients of each herb in the Xiaozheng decoction were collected from the Traditional Chinese Medicine (TCM) database. Network pharmacology was employed to predict the target proteins and pathways of action. Disease databases were utilized to identify target genes associated with bladder cancer. A Protein–Protein Interaction (PPI) network was constructed to illustrate the interaction with intersected target proteins. Key targets were identified using Gene Ontology (GO) and the Kyoto Encyclopedia of Genes and Genomes (KEGG) functional enrichment analysis. A compound-target-pathway network was established after molecular docking predictions. In vitro experiments with bladder cancer cell lines were conducted using core chemical components confirmed by ultra-performance liquid chromatography quadrupole time-of-flight mass spectrometry (UPLC-qTOF-MS) to verify the conclusions of network pharmacology.

**Results:**

45 active compounds were extracted, and their relationships with Traditional Chinese Medicines (TCMs) and protein targets were presented, comprising 7 herbs, 45 active compounds, and 557 protein targets. The intersection between potential TCM target genes and bladder cancer-related genes yielded 322 genes. GO and KEGG analyses indicated that these targets may be involved in numerous cancer-related pathways. Molecular docking results showed that candidate compounds except mandenol could form stable conformations with the receptor. In vitro experiments on three bladder cancer cell lines demonstrated that quercetin and two other impressive new compounds, bisdemethoxycurcumin (BDMC) and kumatakenin, significantly promoted cancer cell apoptosis through the B-cell lymphoma 2/Bcl-2-associated X (Bcl-2/BAX) pathway and inhibited proliferation and migration through the glycogen synthase kinase 3 beta (GSK3β)/β-catenin pathway.

**Conclusion:**

By employing network pharmacology and conducting in vitro experiments, the mechanism of Xiaozheng decoction’s effect against bladder cancer was tentatively elucidated, and its main active ingredients and targets were identified, providing a scientific basis for future research.

## Introduction

Current evidence suggests that bladder cancer was the 10th most prevalent cancer worldwide in 2020 and the most prevalent malignant tumor of the urinary system, responsible for approximately 17,980 cancer-related deaths in the United States [1]. Smoking has been reported to be the most important risk factor for bladder cancer, followed by exposure to petrochemicals. Additionally, the incidence of this disease shows gender-specific differences, with men having approximately four times higher rates than women [2]. Non-muscle-invasive bladder cancer (NMIBC), which remains confined to the epithelium and lamina propria, accounts for over 70% of newly diagnosed cases, while muscle-invasive bladder cancer (MIBC) constitutes 25% of cases [3]. Patients with early-stage NMIBC commonly undergo transurethral bladder resection, and in some cases, intravesical chemotherapy is recommended. However, two-thirds of patients experience relapse within 5 years of treatment, with this proportion rising to 90% after 15 years [4]. Radical cystectomy is invariably required for MIBC patients due to muscle infiltration of the tumor. Despite the availability of multiple therapies such as chemotherapy, radiotherapy, immunotherapy, and targeted therapy for bladder cancer treatment, approximately 50% of MIBC patients experience metastasis within 2 years [5]. Therefore, further research into potential molecular mechanisms and the design of new strategies to improve the survival of bladder cancer patients is crucial.

In Asia, traditional Chinese medicine (TCM) has been widely accepted as a complementary and alternative form of cancer treatment [6]. It is one of the most popular adjuvant therapies for cancer patients after radical surgery in East Asia, offering distinct advantages, including being multi-component, multi-target, and having minimal side effects due to its natural composition, garnering increased attention worldwide. Both retrospective and prospective studies have demonstrated the therapeutic effect of TCM on cancer [7]. Currently, TCM compounds are widely used to treat various cancers and have shown significant therapeutic effects in scientific research and clinical applications, especially against lung cancer [8], breast cancer [9], colorectal cancer [10], and bladder cancer [11]. According to TCM principles, bladder cancer's pathogenesis is attributed to deficiencies of vital qi, dampness, heat, and blood stasis. A well-known empirical prescription from urologist Youfang Liu called Xiaozheng decoction has been reported to prevent the postoperative recurrence of bladder cancer. It comprises *Coix lacryma-jobi* (Yiyiren), *Astragalus membranaceus* (Huangqi), *Polygonatum sibiricum* (Huangjing), *Hedyotis diffusa* (Baihuasheshecao), *Polyporus umbellatus* (Zhuling), *Curcuma phaeocaulis* (Ezhu), and *Rhizoma bolbostemmae* (Tubeimu). Previous clinical trials have demonstrated that the Xiaozheng decoction and bladder perfusion could effectively prolong disease-free survival and control metastasis in bladder cancer patients [12, 13]. In addition to its use in bladder cancer, Xiaozheng decoction has been reported to be effective in treating endometriosis caused by blood stasis [14] and inhibiting the growth of hepatocellular carcinoma by suppressing inflammation [15]. Nonetheless, further research is warranted to elucidate the specific pharmacological actions of Xiaozheng decoction in treating bladder cancer and identify its molecular targets.

**Fig. 1:**
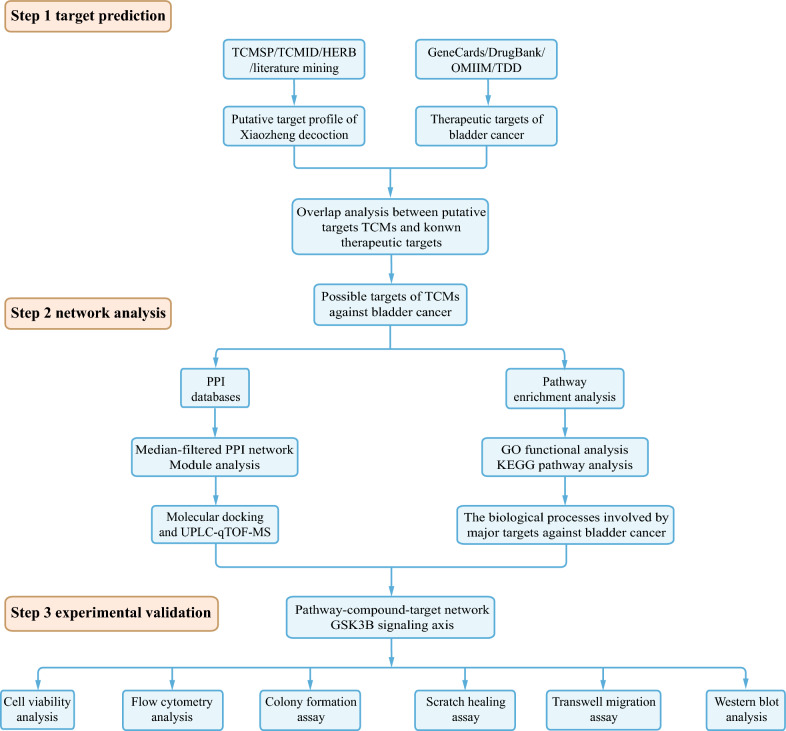
The systematic scheme for investigating the mechanisms of Xiaozheng decoction in the treatment of bladder cancer from network pharmacology to molecular experiments. *TCMSP* Traditional Chinese Medicine System Pharmacology, *TCMID* Traditional Chinese Medicine Information Database, *OMIM* Online Mendelian Inheritance in Man, *TTD* Therapeutic Target Database, *PPI* Protein–Protein Interaction

The past few years have witnessed rapid advancements in systems biology and bioinformatics, facilitating the exploration of TCM from holistic and molecular perspectives. These developments have preliminarily elucidated the molecular mechanisms of active components present in TCM. A novel approach known as network pharmacology has emerged, offering a valuable means to unravel the intricate relationships among compounds, targets, and diseases [16]. This approach aligns with TCM theory, emphasizing the synergy of ingredients and the observation of effects on diseases, providing a systematic perspective for identifying potential drug candidates from extensive translational herbal medicine databases. In this study, we employed network pharmacology to investigate the major components of herbs present in Xiaozheng decoction and identified key compounds and important targets associated with the treatment of bladder cancer. Various bladder cancer cell lines were utilized to verify the pharmacological effects of these key TCM ingredients and potential targets. Figure 1 illustrates the systematic scheme of our study, which encompasses network pharmacology analysis and molecular experiments, aiming to unravel the molecular mechanisms underlying Xiaozheng decoction's efficacy in treating bladder cancer.

## Materials and methods

### Identification of Xiaozheng decoction’s chemical compounds and predictive targets

The chemical ingredients of each herb in Xiaozheng decoction were collected from the Traditional Chinese Medicine System Pharmacology (https://tcmsp-e.com/tcmsp.php, TCMSP) online database and analysis platform, Traditional Chinese Medicine Information Database (https://www.bidd.group/TCMID, TCMID) and the HERB database (http://herb.ac.cn) [17, 18]. To ensure a comprehensive analysis, additional literature mining was performed using the China National Knowledge Internet (https://www.cnki.net, CNKI). Herbal compounds with favorable pharmacological effects were selected as candidate compounds based on specific criteria recommended by TCMSP, which included an oral bioavailability (OB) of ≥ 30% and a drug similarity (DL) of ≥ 0.18. The targets of these selected compounds were obtained from the TCMSP database or predicted using the SwissTargetPrediction platform with a probability threshold of ≥ 0.2 (http://www.swisstargetprediction.ch) [19]. To standardize the data, the targets were normalized using the Universal Protein Resources database (UniProt, https://www.uniprot.org). Cytoscape software was employed to visualize the relationships among the traditional Chinese medicines, potential herbal compounds, and predicted targets.

### Construction of the compound-target-pathway network

Targets associated with human bladder cancer were identified using the search terms “bladder cancer,” “bladder carcinoma,” and “malignant neoplasm of bladder” from (https://www.genecards.org, relevance score ≥ mean) and DrugBank databases (https://go.drugbank.com) with a relevance score greater than or equal to the mean [20–22]. To distinguish between protein-coding and non-coding targets, UniProt was utilized. The intersected targets between the bladder cancer-related targets and potential targets of Xiaozheng decoction were identified using a Venn plot.

The intersected targets from the Venn plot were subjected to analysis using the Search Tool for the Retrieval of Interacting Genes (STRING, https://cn.string-db.org) to investigate the interactions among these protein-coding genes with a confidence level greater than 0.4. The initial protein–protein interaction (PPI) network was further analyzed using Cytoscape along with the CytoNCA plug-in. Nodes with values of intermediate value, compactness, and degree less than their corresponding intermediate values were filtered out. Additionally, the Molecular Complex Detection (MCODE) plug-in was employed to identify and visualize important modules, following specific criteria: degree cutoff = 2, node score cutoff = 0.2, K-core = 2, and maximum depth = 100. Modules with a score of ≥ 10 were selected for further analysis.

Gene Ontology (GO) and Kyoto Encyclopedia of Genes and Genomes (KEGG) pathway analyses were performed by the bioinformatics platform (http://www.bioinformatics.com.cn) to explore the enriched GO terms and KEGG pathways for the identified clusters. Enrichment was based on P values of < 0.01, which aided in predicting the biological processes and mechanisms involved in Xiaozheng decoction’s effects against bladder cancer. The top 25 KEGG pathways and their corresponding targets and compounds were integrated into a compound-target-pathway network using Cytoscape to highlight hub target genes and core compounds.

### Molecular docking

The three-dimensional (3D) structures of the core targets were downloaded from the RCSB Protein Data Bank (RCSB PDB, https://www.rcsb.org), and the 3D structures of potential compounds from PubChem (https://pubchem.ncbi.nlm.nih.gov). AutoDockTools (v1.5.7) was used to remove all water molecules, add hydrogen atoms to the protein structures, and perform protein-compound docking using the Lamarckian genetic algorithm. Subsequently, the docking results were visualized using PyMOL software.

### UPLC-qTOF-MS

The herbs of the Xiaozheng decoction were purchased from Lei Yun Shang Pharmaceutical Group Co. Ltd (Shanghai, China). The Coix lacryma-jobi, Astragalus membranaceus, Polygonatum sibiricum, Hedyotis diffusa, Polyporus umbellatus, Curcuma phaeocaulis, and Rhizoma bolbostemmae were mixed in the ratio of 30:20:20:20:10:10:10 (g). All seven herbs were soaked in deionized water in proportion for one hour. Subsequently, water equal to ten times the weight of the ingredients was added, and the mixture was boiled for an additional 30 min. After decoction and filtration, the filtrate was concentrated to a 1.0 g/mL density and then freeze-dried to obtain powder under vacuum conditions. The resulting powder was stored at – 20 ºC.

Quality control of Xiaozheng decoction was performed by ultra-performance liquid chromatography quadrupole time-of-flight mass spectrometry (UPLC-qTOF-MS) using the Phenomenex Luna C_18_ chromatography column (250 × 4.6 mm, 5 μM) The mobile phase comprised 0.1% formic acid-acetonitrile (A), 0.3% formic acid–water (B). The gradient elution program was as follows: 0–22 min, 4–8% A; 31–60 min, 8–14% A; 65–80 min, 18–25% A; and 85 min, 90% A. The column temperature was set at 35℃, the flow rate was 1 mL/min, the detection wavelength was 250 nm, and the injection volume was 10 μL.

### Cell culture and viability analysis

Human bladder cancer cell lines 5637, J82, and T24, were cultured in RPMI-1640 medium containing 10% fetal bovine serum (FBS; GIBCO, New York, USA) and 1% penicillin–streptomycin (GIBCO, New York, USA) under controlled conditions including a temperature of 37 ℃ and 5%CO_2_. Cell viability was measured by CCK8 (Cell Counting Kit-8; Dojindo, Japan). Briefly, different types of cells (8000/well in 100 μL of medium) were seeded on 96 well plates. After cell attachment, the initial medium was discarded and replaced with the medium with various concentrations (0, 10, 20, 40, 80, 160 μM) of quercetin, bisdemethoxycurcumin (BDMC), and kumatakenin for 24 and 48 h, respectively. Each well was treated with 10 μL CCK8 solution for 2 h. The absorbance of each well was measured at 450 nm using a microplate reader, and the 50% inhibitory concentration (IC_50_) value in each group was calculated by SPSS software.

### Flow cytometry analysis

Cells in the logarithmic phase were inoculated into 6-well plates at a concentration of 1 × 10^5^/mL and incubated overnight. Cells were harvested and resuspended at 1 × 10^6^ cells/mL in phosphate-buffered saline (PBS; GIBCO, New York, USA) after treatment with different concentrations of BDMC and kumatakenin (0, 10, or 20 µM) for 24 h. After two washes in cold PBS, cells were resuspended in PBS containing PI and Annexin V-FITC according to the instructions of the Cell Apoptosis Detection Kit (Dojindo, Japan) and incubated in the dark at room temperature for 30 min. Finally, apoptosis was detected by flow cytometry (BD Biosciences).

### Colony formation assay

J82 and 5637 cells were seeded into 6-well plates at a density of 600 cells/well. Cells were cultured in the completed medium with different concentrations of BDMC and kumatakenin (0, 10, or 20 µM) as determined by cell proliferation measurements. After 10–14 days of incubation, the cell colonies were fixed with 4% paraformaldehyde. The cell colonies were imaged and counted after being stained with 0.1% crystal violet and washed with PBS.

### Scratch healing assay

J82 and 5637 cells were seeded into 6-well plates at a density of 5 × 10^5^ cells/well and allowed to adhere for 24 h to form a monolayer. On the second day, the cell monolayer was scraped from top to bottom with a sterile 200 µL pipette tip, and the cell debris was washed out with PBS. The cells were then incubated in a serum-free RPMI-1640 medium containing BDMC or kumatakenin (0, 10, or 20 µM) for 24 h. An inverted microscope (Nikon, Japan) was used to photograph the cells at 0 and 24 h, and five random visual fields of view were selected (× 40). The healing rate was calculated as follows: scratch healing rate (%) = (A_0−_A_T_)/A_0_ × 100%, where A_0_ was the area of the initial wound, and A_T_ was the remaining area of the wound at that time point.

### Transwell migration assay

After 24 h of serum-free starvation, J82 and 5637 cells were digested and washed twice with PBS, then resuspended in serum-free medium at 3 × 10^5^ cells/mL density. Cells in 200 µL medium containing different concentrations of BDMC and kumatakenin (0, 10, or 20 µM) were inoculated into the upper chamber, and 600 µL medium with 10% FBS was inoculated into the lower chamber in a 24-well plate. After incubation for 8 h, the chambers were fixed with 4% paraformaldehyde and stained with 0.1% crystal violet. After washing with PBS and gently wiping the inside with a cotton swab, the outside of the upper chamber was photographed in five randomly selected fields of view (× 100).

### Western blot analysis

Cells were digested and lysed with ice-cold RIPA lysis buffer (containing 1 mM PMSF). The mixture was then centrifuged at 4 ℃ for 20 min at 12,000 ×*g*. The supernatant protein was collected, and the concentration was determined by bicinchoninic acid assay (BCA). Loading buffer was added to the sample in a ratio of 1:4, and the mixture was boiled for 10 min. The same amount of protein (20 µg) on each sample was separated by 4–15% sodium dodecyl sulfate–polyacrylamide gradient gel electrophoresis (SDS-PAGE) and then transferred to the polyvinylidene fluoride (PVDF) membrane through the transfer box. The PVDF membrane was immersed in the prepared 5% skimmed milk and shaken for 1 h. After being washed, the membrane was placed in a box with the appropriate primary antibody at 4 ℃ overnight. After reacting with the secondary antibody for 1 h, the band was exposed and photographed after dropping the developing solution.

### Statistical analysis

All results were expressed as mean ± standard deviation (SD). Fiji (ImageJ) software was used for image analysis. Data were analyzed using GraphPad Prism 9.0.0, and the significance of the difference was analyzed by one-way analysis of variance analysis. Statistical significance was defined as P < 0.05.

## Results

### The TCM-compound-target network

**Fig. 2:**
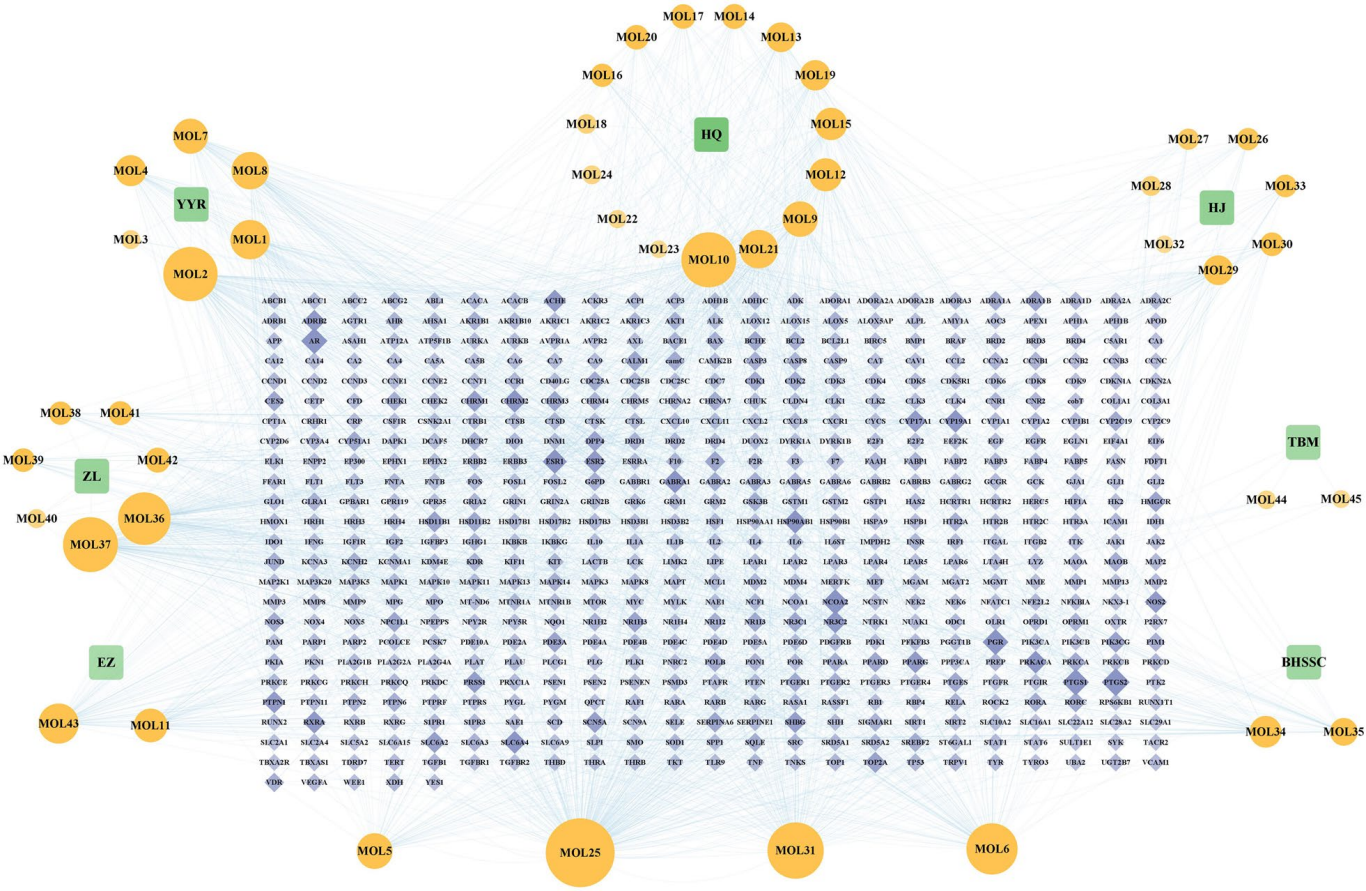
The TCM-compound-target network. *YYR* Yiyiren, *HQ* Huangqi, *HJ* Huangjing, *BHSSC* Baihuasheshecao, *ZL* Zhuling, *EZ* Ezhu, *TBM* Tubeimu

45 active compounds in Xiaozheng decoction were identified, and their interactions with human proteins were screened, as shown in Table 1. After removing duplicates, 557 human proteins were retrieved from databases like TCMSP or predicted by SwissTargetPrediction. Quercetin, kumatakenin, mandenol, polyporusterone G, cerevisterol, and beta-sitosterol emerged as the most important active compounds due to their association with more human protein targets. The relationship between TCMs, compounds, and targets is shown in Fig. 2, encompassing 7 herbs, 45 active compounds, and 557 protein targets. In this network diagram, nodes with higher degree values represented more critical elements, reflected by their larger size and more opaque color. Numerous targets were regulated by various compounds, suggesting that the active components of this prescription act on multiple targets accounting for their efficacy against the disease.

**Table 1:** Information for candidate bioactive compounds retrieved in Xiaozheng decoction

NO	Name	CAS number	MW	OB (%)	DL
*Coix lacryma-jobi* (Yiyiren)					
MOL1	Sitosterol alpha1	474-40-8	426.8	43.28	0.78
MOL2	Mandenol	544-35-4	308.56	42	0.19
MOL3	(6Z,10E,14E,18E)-2,6,10,15,19,23-hexamethyltetracosa-2,6,10,14,18,22-hexaene	7683-64-9	410.8	33.55	0.42
MOL4	[(2R)-2,3-dihydroxypropyl] (Z)-octadec-9-enoate	111-03-5	356.61	34.13	0.3
MOL5	Sitosterol	83-46-5	414.79	36.91	0.75
MOL6	Stigmasterol	83-48-7	412.77	43.83	0.76
MOL7	2-Monoolein	3443-84-3	356.61	34.23	0.29
MOL8	CLR	57-88-5	386.73	37.87	0.68
*Astragalus membranaceus* (Huangqi)					
MOL9	Mairin	472-15-1	456.78	55.38	0.78
MOL10	Kumatakenin	3301-49-3	314.31	50.83	0.29
MOL11	Hederagenin	465-99-6	414.79	36.91	0.75
MOL12	(3S,8S,9S,10R,13R,14S,17R)-10,13-dimethyl-17-[(2R,5S)-5-propan-2-yloctan-2-yl]	64997-52-0	428.82	36.23	0.78
	-2,3,4,7,8,9,11,12,14,15,16,17-dodecahydro-1H-cyclopenta[a]phenanthren-3-ol				
MOL13	Isorhamnetin	480-19-3	316.28	49.6	0.31
MOL14	3,9-di-o-methylnissolin	N/A	314.36	53.74	0.48
MOL15	7-o-methylisomucronulatol	N/A	316.38	74.69	0.3
MOL16	9,10-dimethoxypterocarpan-3-o-β-d-glucoside	94367-42-7	462.49	36.74	0.92
MOL17	(6aR,11aR)-9,10-dimethoxy-6a,11a-dihydro-6H-benzofurano[3,2-c]chromen-3-ol	73340-41-7	300.33	64.26	0.42
MOL18	Bifendate	73536-69-3	418.38	31.1	0.67
MOL19	Formononetin	485-72-3	268.28	69.67	0.21
MOL20	Calycosin	20575-57-9	284.28	47.75	0.24
MOL21	Kaempferol	520-18-3	286.25	41.88	0.24
MOL22	FA	59-30-3	441.45	68.96	0.71
MOL23	Isomucronulatol-7,2ʹ-di-o-glucosiole	N/A	626.67	49.28	0.62
MOL24	1,7-Dihydroxy-3,9-dimethoxy pterocarpene	N/A	314.31	39.05	0.48
MOL25	Quercetin	117-39-5	302.25	46.43	0.28
*Polygonatum sibiricum* (Huangjing)					
MOL26	(2R)-7-hydroxy-2-(4-hydroxyphenyl)chroman-4-one	578-86-9	256.27	71.12	0.18
MOL27	DFV	578-86-9	256.27	32.76	0.18
MOL28	4ʹ,5-Dihydroxyflavone	6665-67-4	254.25	48.55	0.19
MOL29	Baicalein	491-67-8	270.25	33.52	0.21
MOL30	3ʹ-Methoxydaidzein	21913-98-4	284.28	48.57	0.24
MOL5	Sitosterol	64997-52-0	414.79	36.91	0.75
MOL31	Beta-sitosterol	83-46-5	414.79	36.91	0.75
MOL32	( +)-Syringaresinol-o-beta-d-glucoside	7374-79-0	580.64	43.35	0.77
MOL33	Diosgenin	512-04-9	414.69	80.88	0.81
*Hedyotis diffusa* (Baihuasheshecao)					
MOL34	Poriferasterol	481-16-3	412.77	43.83	0.76
MOL35	2-methoxy-3-methyl-9,10-anthraquinone	17241-42-8	252.28	37.83	0.21
MOL6	STIGMASTEROL	83-48-7	412.77	43.83	0.76
MOL31	Beta-sitosterol	83-46-5	414.79	36.91	0.75
MOL25	Quercetin	117-39-5	302.25	46.43	0.28
*Polyporus umbellatus* (Zhuling)					
MOL36	Polyporusterone G	141360-94-3	458.75	33.43	0.81
MOL37	Cerevisterol	516-37-0	430.74	37.96	0.77
MOL38	(22e,24r)-ergosta-7,22-dien-3-one	N/A	396.72	44.88	0.72
MOL39	Ergosta-7,22-dien-3-one	N/A	396.72	44.88	0.72
MOL40	Ergosta-5,7,22-trien-3-ol	N/A	396.72	46.18	0.72
MOL41	Ergosta-7,22E-dien-3beta-ol	2465-11-4	398.74	43.51	0.72
MOL42	Ergosta-7,22-diene-3β-ol	N/A	398.74	43.51	0.72
*Curcuma phaeocaulis* (Ezhu)					
MOL11	Hederagenin	465-99-6	414.79	36.91	0.75
MOL43	Bisdemethoxycurcumin	24939-16-0	308.35	77.38	0.26
*Rhizoma Bolbostemmae* (Tubeimu)					
MOL44	Δ7,16,25,26-stigmastatrienol	N/A	410.75	46.21	0.76
MOL45	Δ7,22,25-triene-3-ol	N/A	410.75	46.67	0.76
MOL5	Sitosterol	64997-52-0	414.79	36.91	0.75
MOL31	Beta-sitosterol	83-46-5	414.79	36.91	0.75

*MW* molecular weight, *OB* oral bioavailability, *DL* drug-likeness

### The compound-target-pathway network

**Fig. 3:**
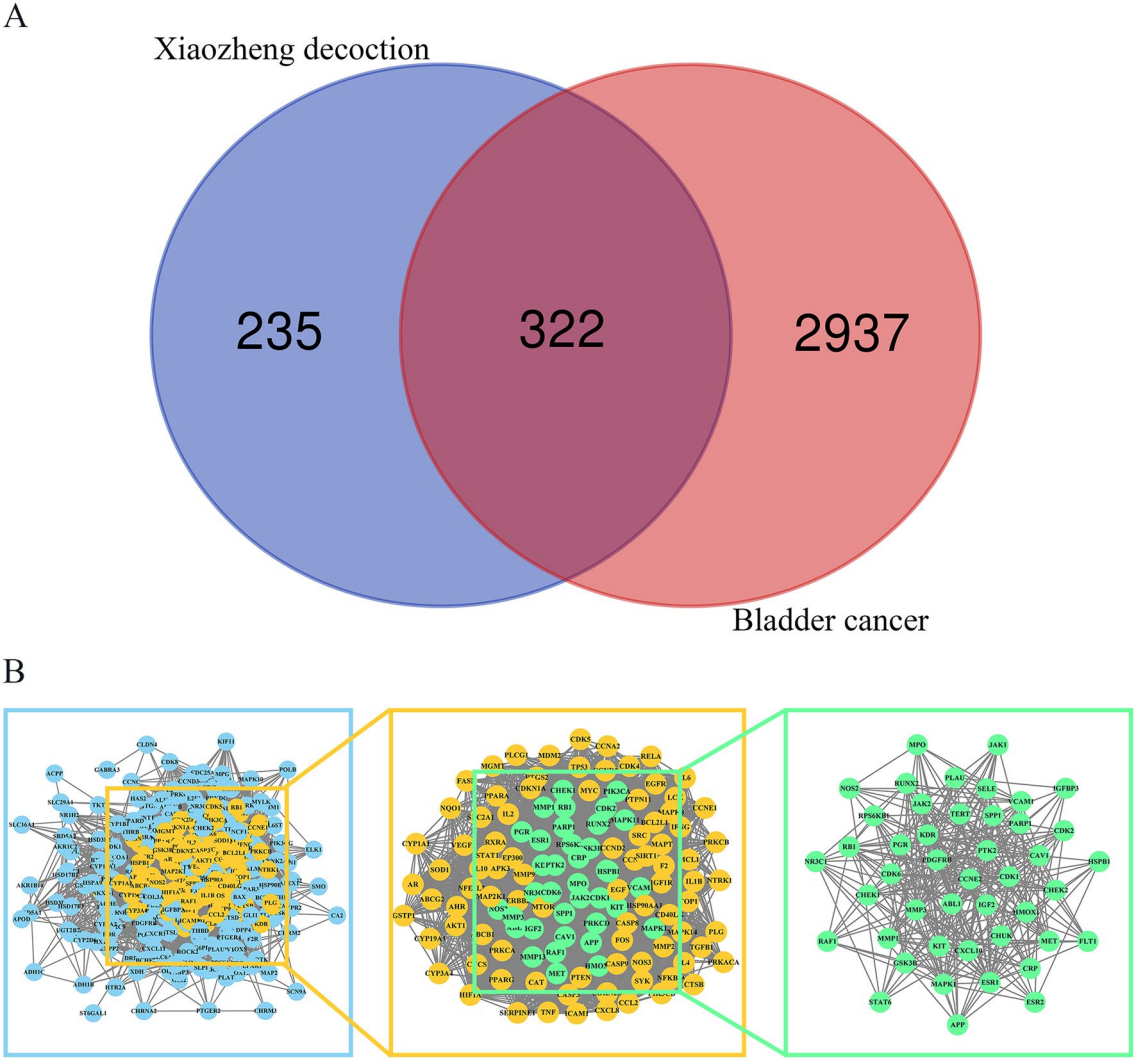
The process of discovering key intersected genes. The Venn plot of Xiaozheng decoction and bladder cancer (**A**). The PPI network analysis of the targets (**B**)

A total of 3259 human protein-coding genes related to bladder cancer were extracted from the databases using a correlation score greater than the median. The Venn plot in Fig. 3A illustrates 322 genes that overlap between the potential target genes of Xiaozheng decoction and the genes related to bladder cancer, thus providing insights into the therapeutic pathway of Xiaozheng decoction for treating bladder cancer.

Subsequently, we conducted a PPI network analysis on the candidate target genes using the STRING website. This analysis yielded a primary PPI network of 320 nodes and 8136 edges, with 2 unconnected genes discarded. Figure 3B illustrates the first screening process, where we applied criteria such as betweenness, closeness, and degree to establish a core network with 125 nodes, all of which had three parameters greater than the median. For the second screening, we employed the MCODE plug-in, resulting in the selection of a module with the highest score, comprising 45 nodes.

**Fig. 4:**
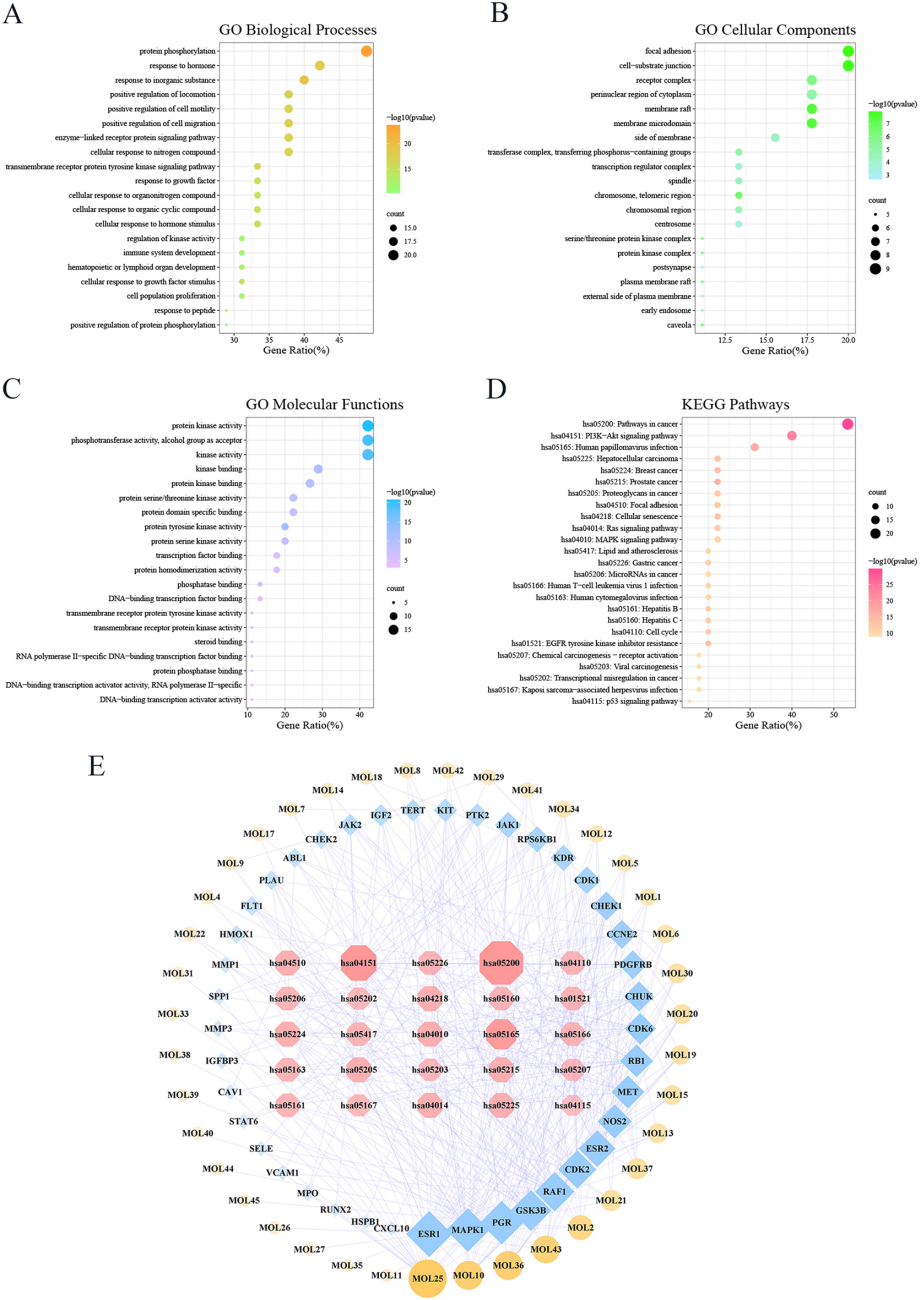
The construction of the compound-target-pathway network. Analyses of key intersection genes for the top 20 GO annotations of biological processes (**A**), cellular components (**B**), and molecular functions (**C**) and the top 25 KEGG pathways (**D**). The compound-target-pathway network for the top 25 KEGG pathways (**E**)

To gain insights into the functions of these protein targets, we performed functional enrichment analyses using GO and KEGG databases. The results revealed that the key targets were closely associated with processes such as protein phosphorylation, positive regulation of locomotion, cell motility, and cell migration (Fig. 4A). The GO cellular components analysis highlighted focal adhesion, cell-substrate junction, and membrane raft as important aspects (Fig. 4B). Additionally, GO molecular functions indicated that the genes primarily played roles in protein kinase activity, phosphotransferase activity, and kinase binding (Fig. 4C). KEGG pathway analysis suggested that these targets might be involved in pathways such as pathways in cancer, PI3K/Akt signaling pathway, focal adhesion, and proteoglycans in cancer (Fig. 4D), implying that the composite targets were clustered in similar functional pathways.

We constructed a compound-target-pathway network to visually represent the potential targets of the compounds and their corresponding signaling pathway mechanisms (Fig. 4E). Notably, the highest degree values were observed for estrogen receptor 1 (ESR1), mitogen-activated protein kinase 1 (MAPK1), glycogen synthase kinase 3 beta (GSK3β), Raf-1 proto-oncogene (RAF1), and cyclin-dependent kinase 2 (CDK2), indicating their significance as central targets in the network.

### Molecular docking and UPLC-qTOF-MS

**Fig. 5:**
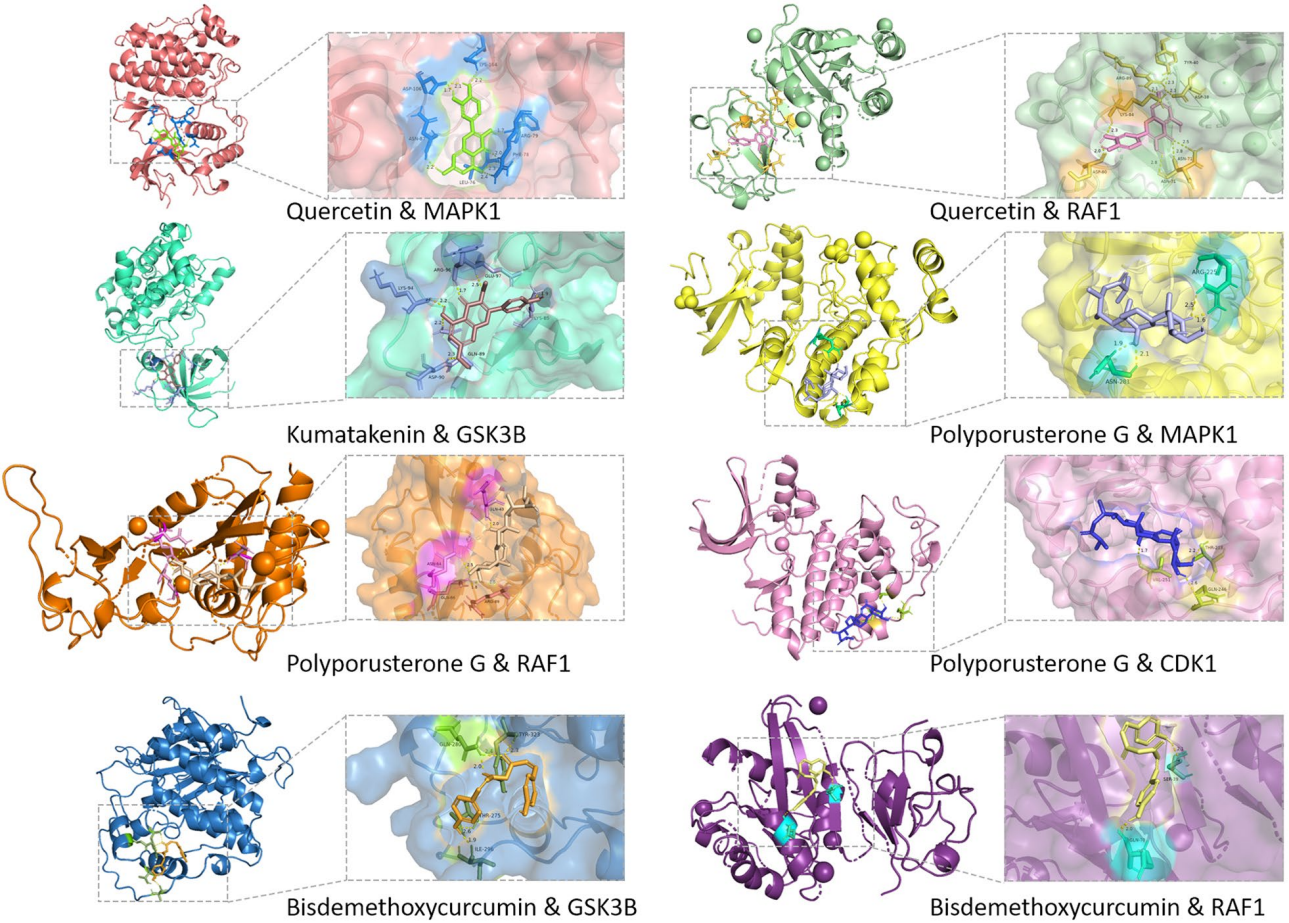
The conformations of main active compounds and major targets with binding affinity ≤ − 6.5 kcal/mol

AutoDockTools was employed for molecular docking to explore the interactions between essential active compounds and primary target proteins. Figure 4E portrays the top five key compounds (quercetin, kumatakenin, polyporusterone G, BDMC, and mandenol) and their docking attempts with five hub target proteins (ESR1, MAPK1, GSK3β, RAF1, CDK2). The binding energies of 25 dockings were recorded and presented in Table 2, indicating the affinity strength. Notably, stable conformations with ≤ − 6.5 kcal/mol binding affinities were formed between key active compounds and major hub target molecules (Fig. 5), except for mandenol.

**Table 2:**
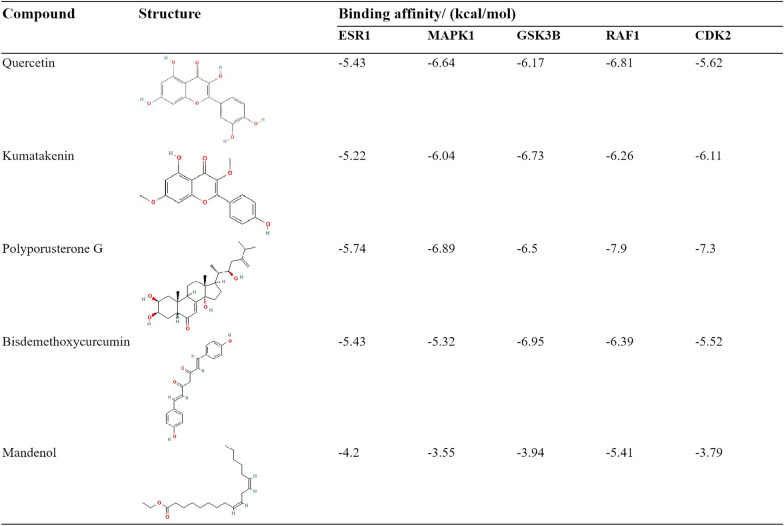
The structure of the core compounds and binding energy between compounds and targets

**Fig. 6:**
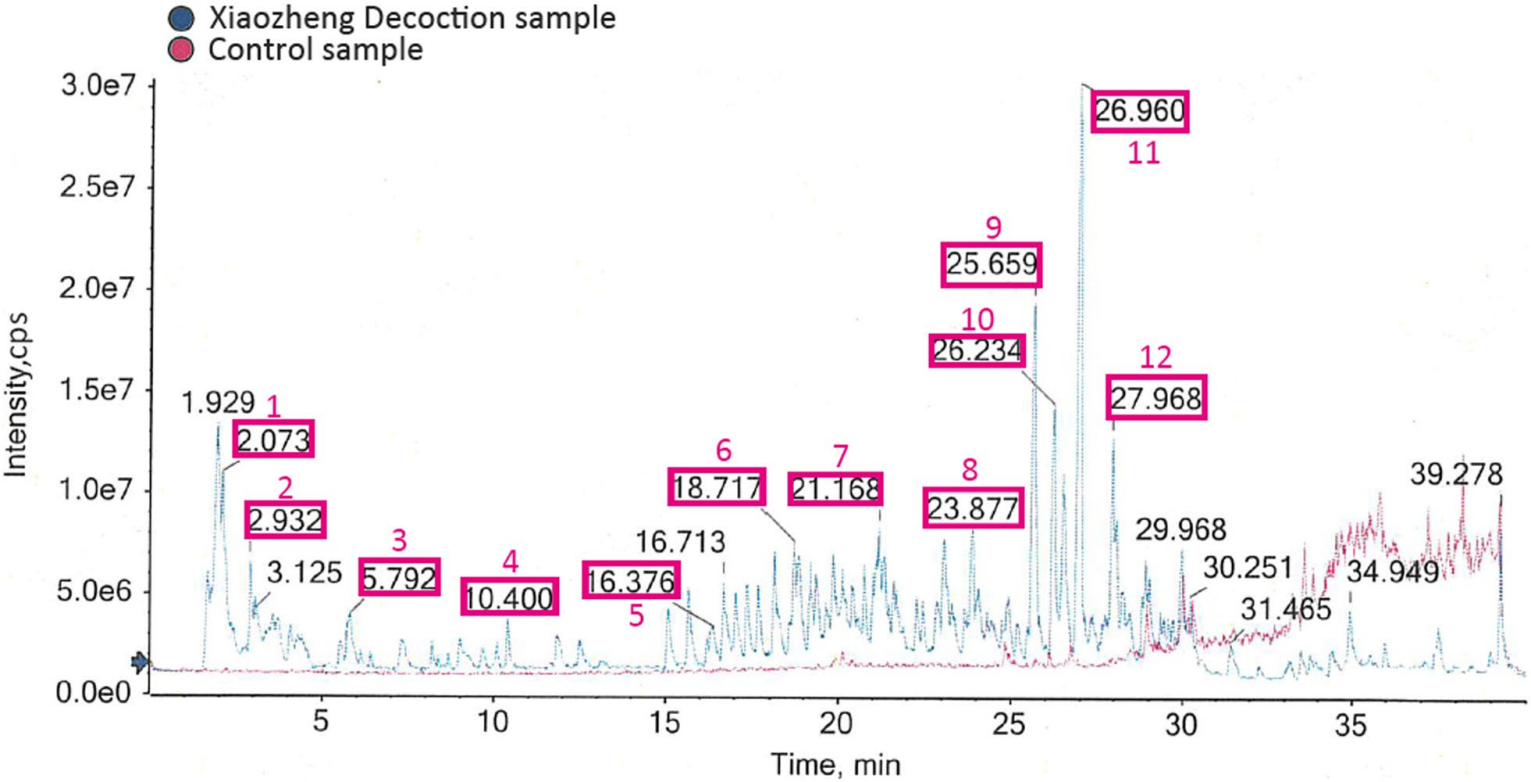
Total ion chromatograms of Xiaozheng decoction by UPLC-Q-TOF–MS and its representative compounds: 1. Polyporusterone G; 2. Curcumenol; 3. Curdione; 4. Atractylenolide I; 5. Wogonin; 6. Quercetin; 7. Kumatakenin; 8. Bisdemethoxycurcumin; 9. Stigmasterol; 10. β-Sitosterol; 11. Sitosterol; 12. Mandenol

To determine the major components and ensure quality control in Xiaozheng decoction, we utilized UPLC/Q-TOF–MS/MS in our analysis, as shown in Fig. 6.

### Xiaozheng decoction inhibited the proliferation of bladder cancer cells and two components played a vital role

**Fig. 7:**
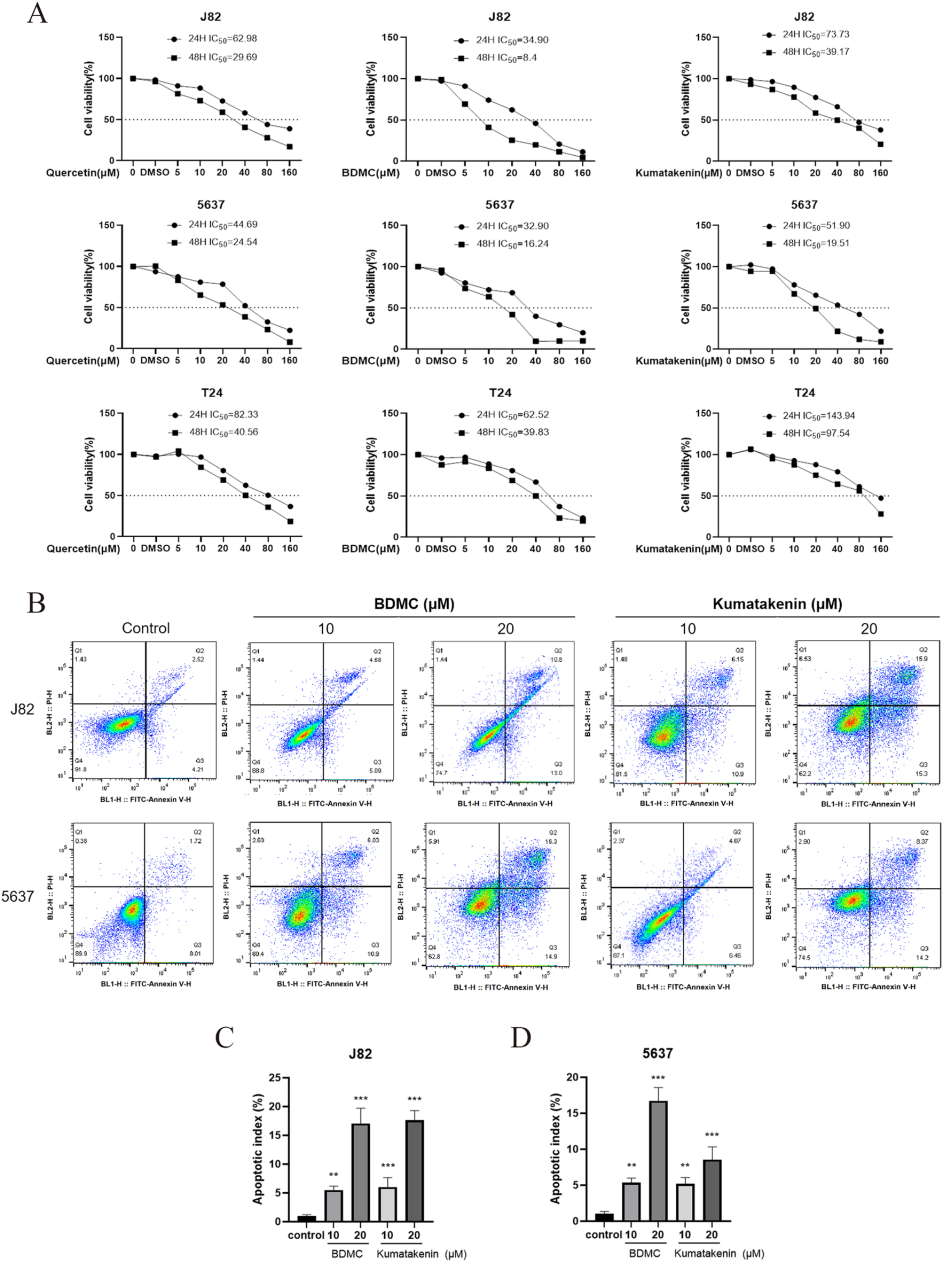
The compounds in Xiaozheng decoction inhibited the proliferation of bladder cancer cells. Drug concentration-cell viability curves were generated based on the CCK-8 assays (**A**). Annexin V-FITC/PI staining assay for analysis of apoptotic cell death (**B**) in J82 (**C**) and 5637 cells (**D**). **P < 0.01, ***P < 0.001 versus control (−) group; n = 5

To validate the bioinformatics results, cell culture evaluation was conducted to assess the pharmacological effects of the main components. Five main compounds were identified: quercetin, kumatakenin, polyporusterone G, BDMC, and mandenol. However, due to challenges in obtaining or accurately extracting polyporusterone G with high purity and weak docking of mandenol with the target molecule of the disease, these two compounds were excluded from the analysis. Accordingly, the effects of quercetin, kumatakenin, and BDMC on the proliferation of human bladder cancer cell lines J82, 5637, and T24 were investigated. The IC_50_ values of quercetin, kumatakenin, and BDMC at 48 h on J82 cells were 29.69, 8.4, and 39.17, respectively, lower than the IC_50_ values at 24 h, suggesting a decrease in IC50 values over time. Additionally, quercetin, kumatakenin, and BDMC exhibited stronger cytotoxicity on J82 and 5637 cells compared to T24 cells at relatively low concentrations. The cell viability curve is shown in Fig. 7A.

While the pharmacological effects of quercetin on bladder cancer have been widely researched [23, 24], our focus was on investigating the other two compounds (kumatakenin and BDMC), which showed efficacy comparable to quercetin. PI and Annexin V-FITC double staining was performed to assess whether cell death induced by the compounds was related to apoptosis. Flow cytometry results (Fig. 7B) indicated that treatment with kumatakenin and BDMC significantly increased the number of early and late apoptotic cells in J82 and 5637 cells in a concentration-dependent manner (P < 0.05). These findings (Fig. 7C and D) suggested that BDMC and kumatakenin could induce apoptosis in human bladder cancer cells, accounting for their observed effects on cell viability.

**Fig. 8:**
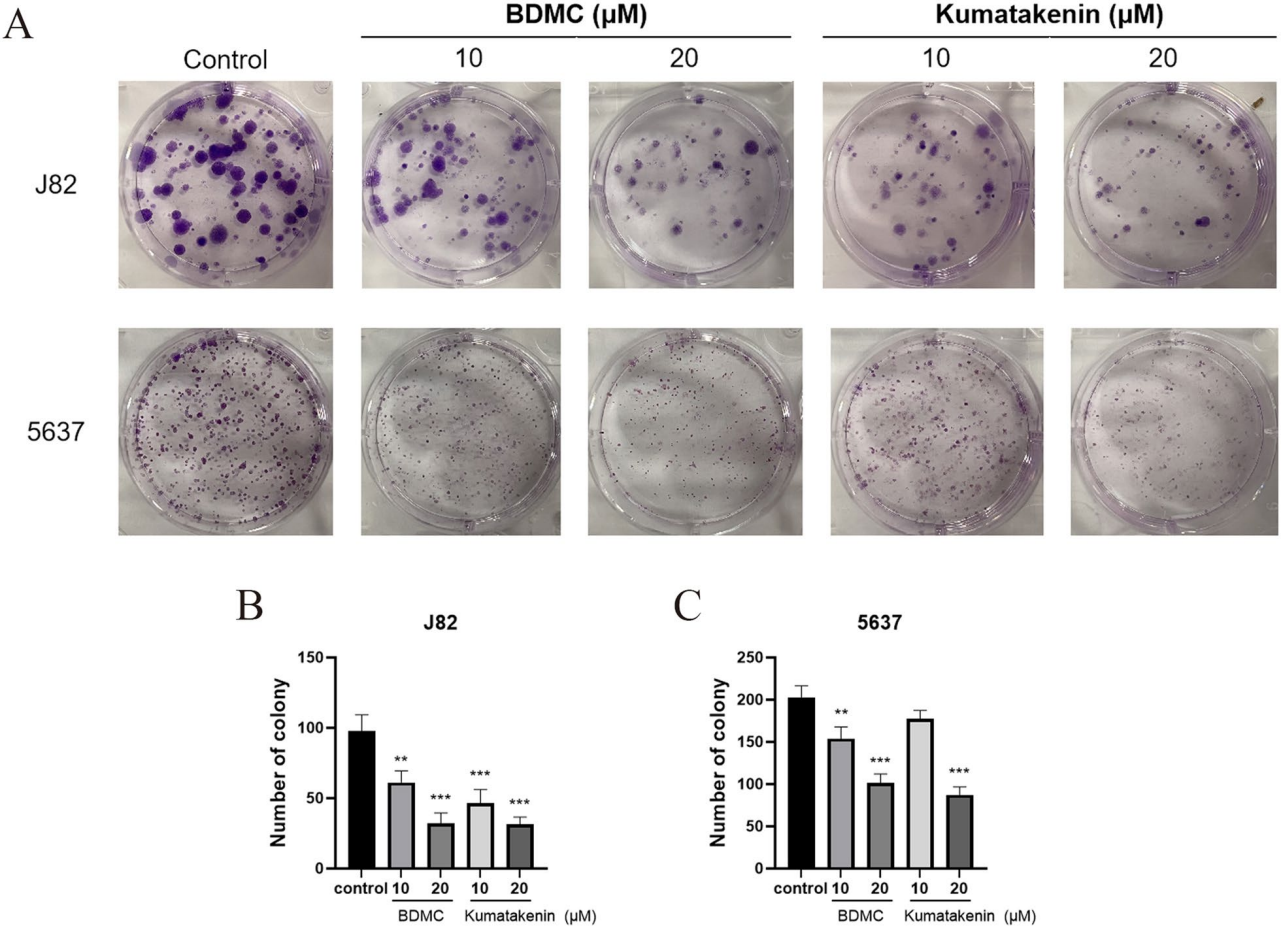
The compounds in Xiaozheng decoction inhibited the colony formation of bladder cancer cells (**A**). The number of colonies was significantly decreased by the treatment with two compounds in J82 (**B**) and 5637 cells (**C**). **P < 0.01, ***P < 0.001 versus control (−) group; n = 5

Colony formation assays were conducted to determine the antiproliferative effect of BDMC and kumatakenin. The results (Fig. 8) showed that treatment with BDMC and kumatakenin significantly inhibited colony formation ability compared to the DMSO group (P < 0.01), indicating their ability to suppress the proliferation of bladder cancer cells.

### BDMC and kumatakenin attenuated migration in bladder cancer cells in a dose-dependent manner

**Fig. 9:**
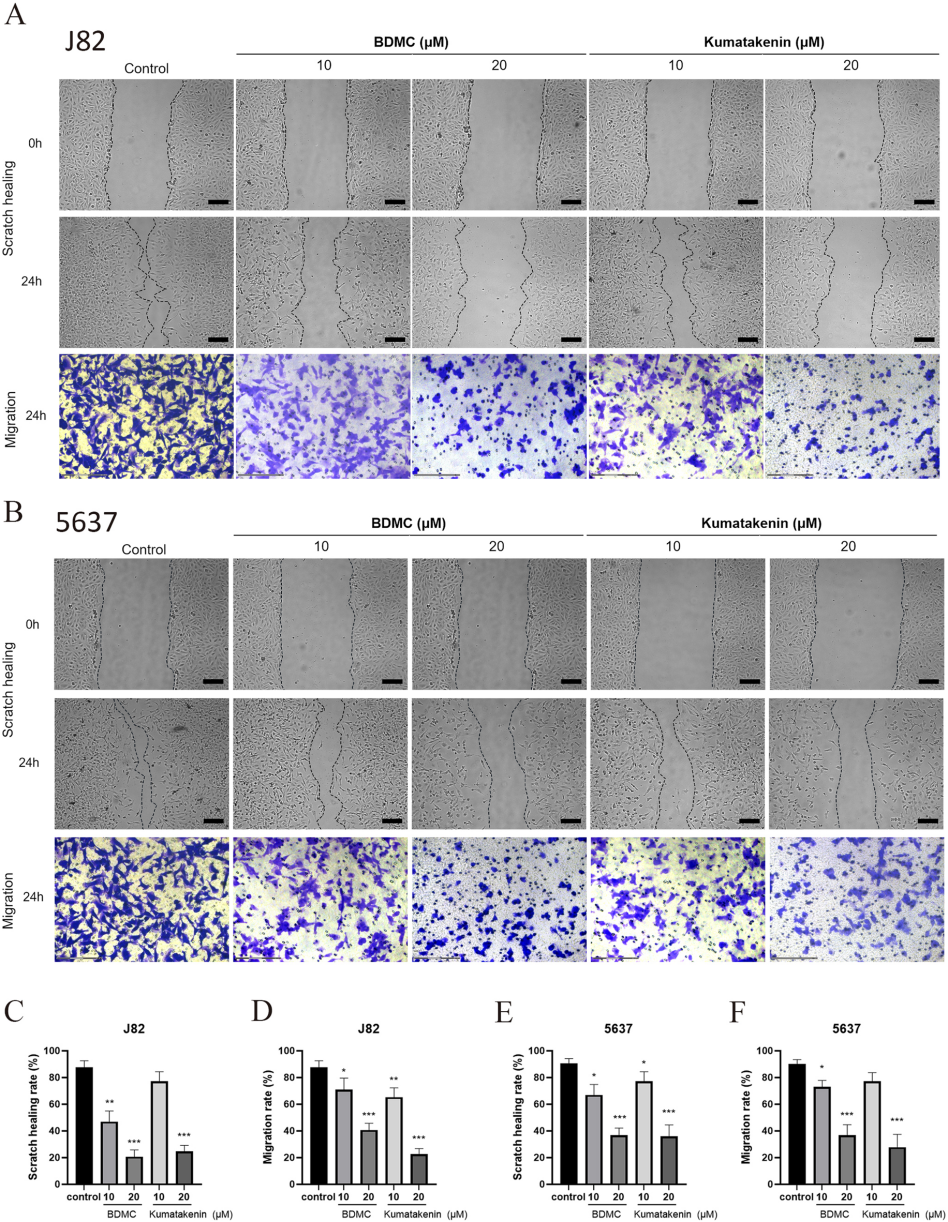
The compounds in Xiaozheng decoction inhibited the healing and migration abilities of bladder cancer cells (**A**, **B**). The healing rate and migration rate were significantly decreased by the treatment of two compounds in J82 (**C**, **D**) and 5637 cells (**E**, **F**). *P < 0.05, **P < 0.01, ***P < 0.001 versus control (−) group; n = 5

To investigate the potential anti-metastatic effect of BDMC and kumatakenin on bladder cancer cells, wound healing and Transwell migration assays were performed on J82 and 5637 cells. Scratch assays (Figs.  9A and 9B) showed that the treatment with BDMC and kumatakenin significantly prolonged the healing process of J82 and 5637 cells in a concentration-dependent manner compared to the control group (P < 0.01; Figs.  9C and 9D). Moreover, BDMC and kumatakenin significantly reduced the number of J82 and 5637 cells invading the lower chamber of Transwell in response to FBS stimulation (P < 0.01; Figs.  9E and 9F), indicating their effective inhibitory effect against bladder cancer invasion.

### BDMC and kumatakenin promoted apoptosis and inhibited migration through the GSK3β/β-catenin pathway in bladder cancer

**Fig. 10:**
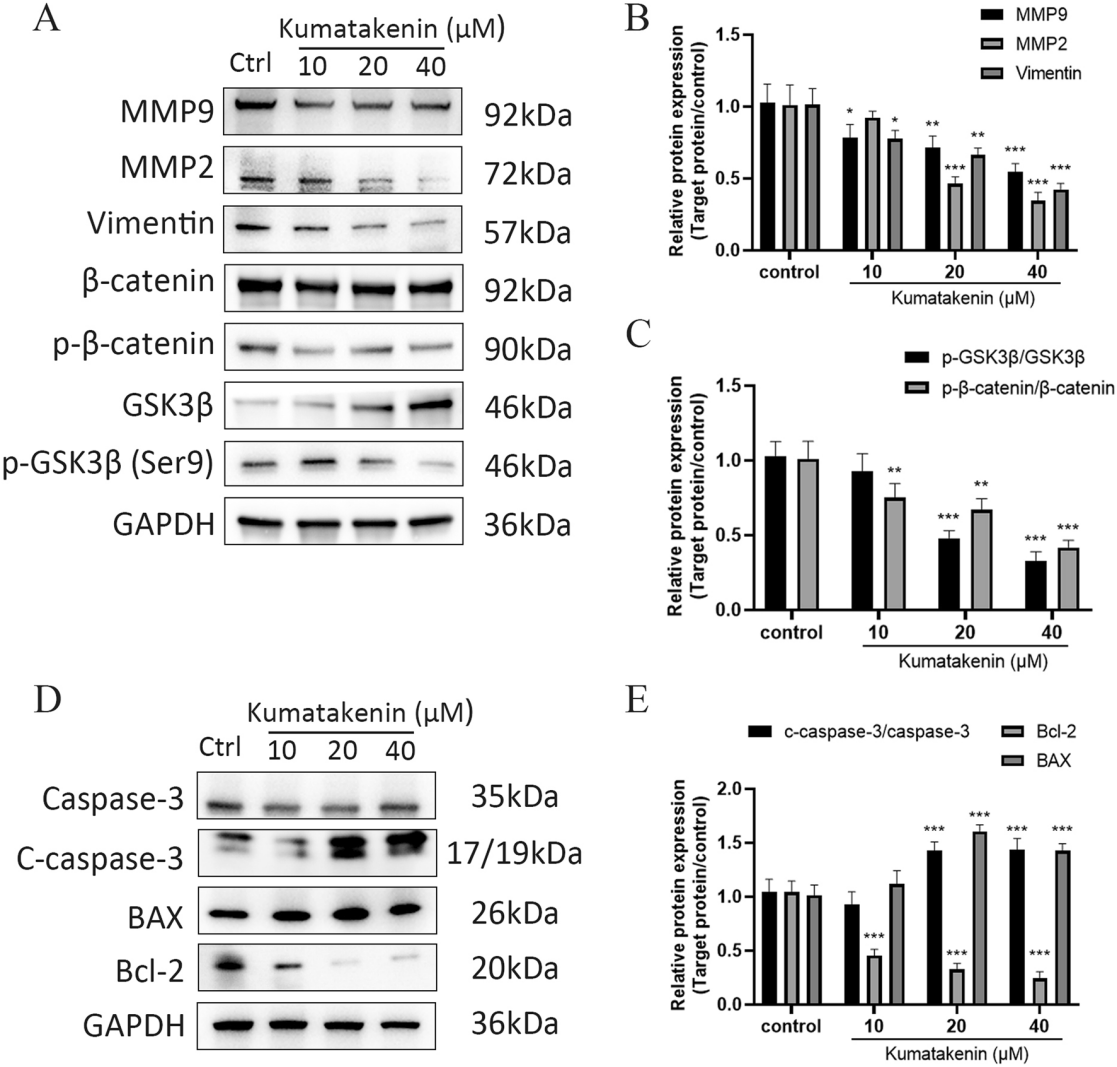
BDMC regulated the GSK3β/β-catenin and apoptosis signaling pathway in J82 cells. MMP9, MMP2, vimentin, and GSK3β/β-catenin expression in J82 cells under BDMC treatment (**A**–**C**). The expression of caspase-3 and Bcl-2/BAX in J82 cells under BDMC treatment (**D**, **E**). Representative blots are presented with the densitometry results; GAPDH served as a control. Values are expressed as the mean ± standard deviation of five independent experiments. **P < 0.01 and ***P < 0.001, versus control group. BDMC and kumatakenin promoted apoptosis and inhibited the migration via the GSK3β/β-catenin pathway in bladder cancer

**Fig. 11:**
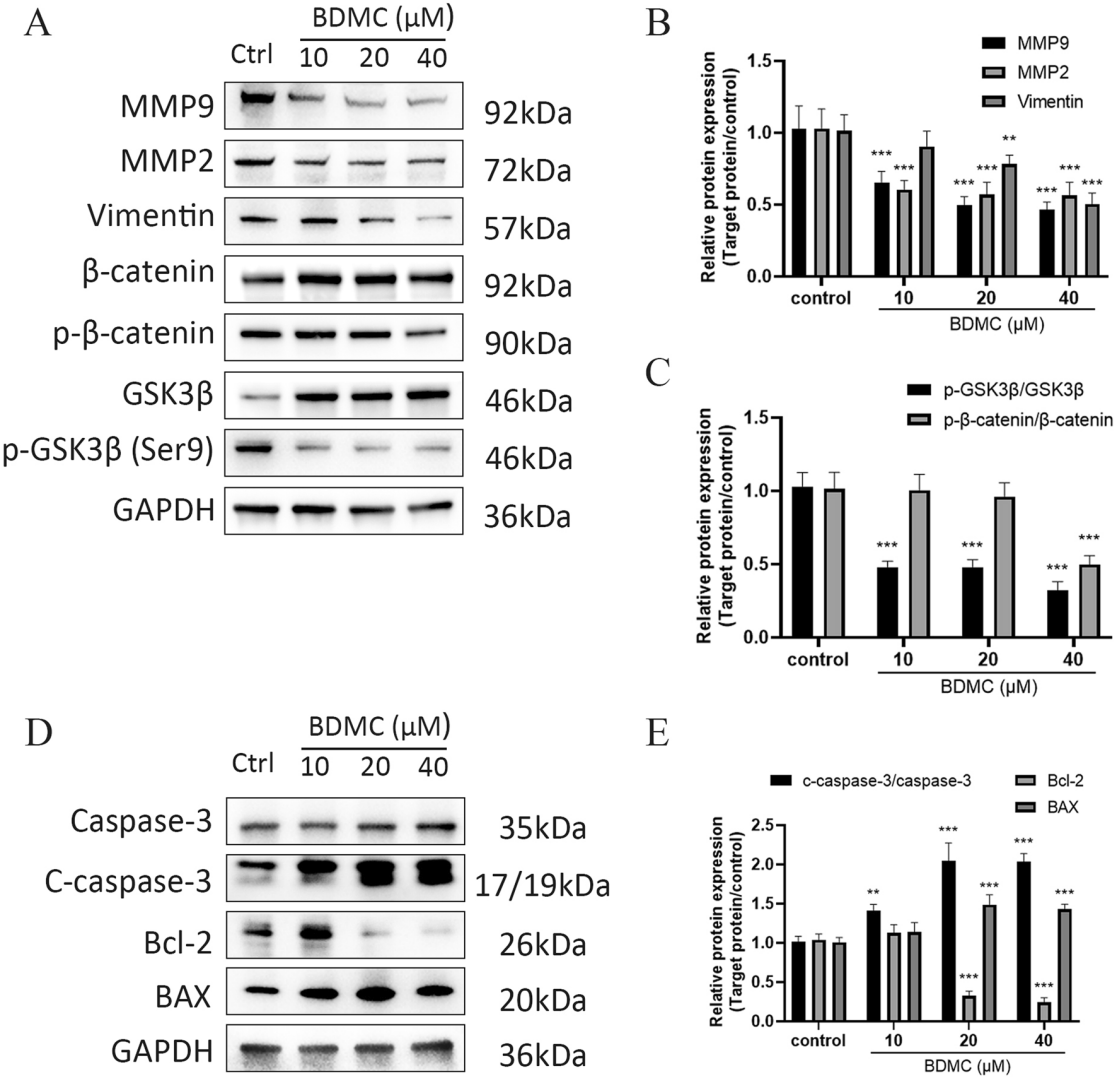
Kumatakenin regulated the GSK3β/β-catenin signaling pathway in J82 cells. The expression of MMP9, MMP2, vimentin, and GSK3β/β-catenin in J82 cells under kumatakenin treatment (**A**–**C**). The expression of caspase-3 and Bcl-2/BAX in J82 cells under kumatakenin treatment (**D**, **E**). Representative blots are presented with the densitometry results; GAPDH served as a control. Values are presented as the mean ± standard deviation of five independent experiments. *P < 0.05 **P < 0.01 and ***P < 0.001, versus control group

Western blot assays were performed to verify the signaling pathway of apoptosis found by flow cytometric analysis induced by BDMC and kumatakenin. Apoptosis is a type of programmed cell death dependent on caspases and regulated by the B-cell lymphoma 2/Bcl-2-associated X (Bcl-2/BAX) signaling pathway. The results (Figs. 10D and 11D) showed that BDMC and kumatakenin decreased the expression of Bcl-2 and increased the expression of c-caspase-3 and Bax in J82 cells in a concentration-dependent manner compared to the control group (P < 0.01; Figs. 10E and 11E). These findings suggested that Bcl-2/Bax signaling pathway regulation by these two compounds might contribute to apoptosis in J82 cells.

The expression of metastasis-associated proteins was measured by Western blot analysis. As shown in Figs. 10A and 11A, the mesenchymal marker vimentin was significantly downregulated, and the levels of MMP2 and MMP9 were significantly reduced in the J82 cells treated with BDMC and kumatakenin compared to the control group (Figs. 10B and 11B). Taken together, these results indicate that BDMC and kumatakenin exerted anti-metastatic effects by suppressing the epithelial-mesenchymal transition (EMT) process and the secretion of MMPs. To further elucidate the possible regulatory mechanisms, we primarily explored the GSK3β/β-catenin signaling pathway based on the molecular docking result. The results demonstrated that the two components significantly inhibited the phosphorylation at the Ser9 site of GSK3β, thereby inducing the degradation of phosphorylated β-catenin (Figs. 10C and 11C; P < 0.01). These findings suggest that the GSK3β / β-catenin pathway may play a role in reducing cell metastasis induced by these compounds.

## Discussion

**Fig. 12:**
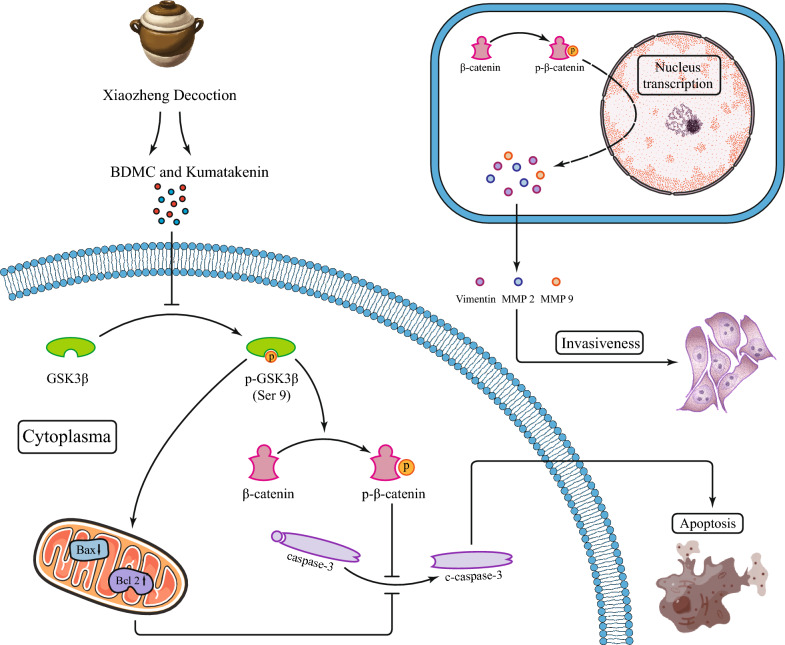
Xiaozheng decoction achieves anti-tumor effects against bladder cancer via the GSK3β/β-catenin signaling pathway

It is well-established that bladder cancer is one of the most common malignant tumors. Transurethral resection of bladder tumor (TURBT) is an important diagnostic and therapeutic method for NMIBC. Although postoperative intravesical instillation can effectively reduce the recurrence and has become the standard treatment for low-risk to high-risk NMIBC after TURBT, significant limitations remain for high-risk NMIBC patients with high risks of recurrence and disease progression after discharge. Indeed, MIBC is a life-threatening disease that affects patients worldwide. Although neoadjuvant therapy is effective, novel bladder cancer drugs are still needed for patients with limited responses to existing drugs. TCM holds unique strengths in utilizing natural products for tumor treatment, and some of these approaches have already found their way into clinical applications, indicating the potential for TCM to serve as a valuable source for the design of novel anti-tumor drugs in the future. Certain TCM formulations have been proven effective in treating bladder cancer, and network pharmacology has played a crucial role in uncovering the specific targets and mechanisms responsible for their therapeutic actions [25, 26]. Xiaozheng decoction is a famous TCM prescription for bladder cancer in the clinic, which consists of 7 herbs. Pharmacological studies have shown that Xiaozheng decoction could improve anti-inflammatory capacity and regulate lipid-related metabolism to combat liver cancer and induce autophagy in prostate cancer cells [27, 28]. Although the effect of Xiaozheng decoction on bladder cancer has been established in clinical practice [13], its specific mechanism has not been clarified. To address this gap, we conducted an experimental investigation guided by network pharmacology to explore and elucidate the mechanisms underlying its therapeutic effects (Fig. 12).

According to our results, 45 active compounds in Xiaozheng decoction and 322 targets overlapping with bladder cancer were identified, indicating that this formulation exerts a pharmacological effect on bladder cancer through multiple targets. Quercetin, kumatakenin, polyporusterone G, BDMC, and mandenol were identified as the top five important active compounds. Quercetin is a type of flavonoid with multiple biological activities widely distributed in the plant kingdom. It has been shown to induce apoptosis of bladder cancer cells through the AMPK pathway [29] and yield a sensitizing effect for bladder cancer radiotherapy [30]. Kumatakenin is a natural product first documented in clove, which could induce apoptosis of ovarian cancer cells and inhibit the expression of M2 markers to regulate the tumor microenvironment [31]. Polyporusterone G, a compound belonging to the family of compounds found in *Polyporus umbellatus*, has been recognized for its cytotoxic properties on leukemia cell proliferation [32]. Besides, *Polyporus umbellatus* has been shown to promote apoptosis by downregulating AKT in breast cancer [33]. BDMC, a lipid-soluble polyphenolic curcuminoid, yields a range of biological activities, including cytotoxicity in various human cancer cell types, and is more stable than curcumin [34, 35]. Mandenol has been identified as a long-chain fatty acid ethyl ester that acts as a plant metabolite and an anti-inflammatory agent to combat cancer cells [36]. In short, Xiaozheng decoction is a multi-component prescription with multi-target efficacy, and the relationship between these agents and bladder cancer warrants further investigation.

Five hub target proteins and their related pathways involved in bladder cancer were screened from the compound-target-pathway network analysis. Among these hub targets, ESR1 emerged as the most significant one. Several studies have validated the essential role of ESR1 in bladder cancer risk stratification [37, 38], especially at the tumor grade level [39], though this cancer is not typically regarded as hormone-related. However, upon conducting molecular docking experiments, the affinity of ESR1 was found to be less than ideal, leading us to discontinue further validation for this target. Similarly, the progesterone receptor (PGR), another hormone-related target, was not considered for further validation due to the minimal expression of this protein in bladder cancer [39, 40].

Consistent with the KEGG pathway analysis results, our hub targets showed significant involvement of MAPK signaling-related proteins, particularly MAPK1 and RAF1. The MAPK signaling pathway is a three-tiered signaling cascade composed of RAF, MEK, and ERK, which are serine/threonine-specific kinases that regulate critical cellular processes, such as cell proliferation, differentiation, and migration [41–43]. Based on the pharmacological effects observed when blocking RAF1, it is suggested that quercetin and BDMC, derived from Xiaozheng decoction, may play a crucial role in inhibiting RAF1/MAPK-dependent malignant biological behavior in bladder cancer cells. Both quercetin and BDMC displayed cytotoxic effects in all three bladder cancer cell lines, as demonstrated by the CCK8 assay. Furthermore, BDMC induced cell apoptosis in a dose-dependent manner and exhibited inhibitory effects on colony formation and cell invasion. Another significant target of interest is CDK2, a member of the serine/threonine protein kinases family involved in cell cycle regulation, crucial for driving cell progression through the S- and M-phases of the cell cycle [44]. Interestingly, numerous studies have reported that phytotherapy exerts anti-cancer effects via CDK2-mediated pathways. For example, genistein, an isoflavone derived from soy, was found to promote apoptosis induction in T24 cells, associated with G2/M phase cell cycle arrest and CDK2 inhibition through regulation of the ROS-dependent PI3K/Akt signaling pathway [45]. Similarly, licochalcone A demonstrated a similar mechanism of ROS-mediated cell cycle arrest and apoptosis, involving CDK2 blockade [46]. In the present study, during virtual docking analysis of Xiaozheng decoction compounds, polyporusterone G exhibited the highest binding affinity to CDK2. However, due to the limited accessibility of polyporusterone G, we could not validate the cell cycle arrest effect of this rare compound derived from Xiaozheng decoction.

GSK3β, another type of serine/threonine protein kinase, was first described as a component of glycogen synthase regulation via its phosphorylation. As a core downstream component of PI3K/Akt [47], GSK3β mediates many biological processes in tumor cells and promotes the development and metastasis of many types of tumors through EMT [48, 49]. One study has demonstrated that GSK3β is involved in the β-catenin/Snail1 pathway to promote the EMT process in bladder cancer [50]. Moreover, targeting GSK3β with 9-ING-41 (a small molecule inhibitor of GSK3β) has been observed to have multiple anti-bladder cancer effects, including cell cycle arrest, autophagy, and apoptosis in bladder cancer cells. Combined with gemcitabine or cisplatin, 9-ING-41 enhanced the growth inhibitory effects [51]. Our study found that kumatakenin and BDMC have huge potential as GSK3β inhibitors, exhibiting effective inhibition of tumor cell growth. Furthermore, these compounds demonstrated the ability to suppress cell migration and invasion. Western blot analysis revealed that kumatakenin and BDMC dose-dependently inhibited the phosphorylation of GSK3β and β-catenin. The impairment of cell invasion ability was mechanistically elucidated by the downregulation of MMP9, MMP2, and vimentin upon treatment with kumatakenin or BDMC through the EMT process. Flow cytometry and Western blot analysis revealed pro-apoptotic effects of GSK3β inhibition, accompanied by downregulation of the Bcl-2/BAX ratio, indicating the induction of apoptosis.

## Conclusion

By employing network pharmacology and conducting in vitro experiments, we have gained preliminary insights into the mechanism through which Xiaozheng decoction exerts its effects against bladder cancer (Fig. 12). Moreover, this approach allowed us to identify the primary active ingredients and their respective targets, establishing a scientific foundation for further research endeavors.

## Data Availability

The datasets presented in this study can be found in online repositories. The names of the repository/repositories and accession number(s) can be found in the article.
